# Epidemiological and biological characteristics of IncR plasmids as multihost antibiotic resistance carriers

**DOI:** 10.3389/fmicb.2025.1622352

**Published:** 2025-07-17

**Authors:** Duo Zhang, Shuang Li, Xindan Zhang, Shuai Zheng, Di Zhou, Qinlong Hou, Gen Li, Huiming Han

**Affiliations:** ^1^The School of Basic Medicine, Beihua University, Jilin, China; ^2^The Center for Infection and Immunity, Beihua University, Jilin, China; ^3^Department of Science and Education, Affiliated Hospital of Beihua University, Jilin, China; ^4^The School of Stomatology, Beihua University, Jilin, China

**Keywords:** IncR plasmid, epidemiology, antimicrobial resistance, co-linear plasmids, inter-host transmission

## Abstract

IncR plasmids are important resistance vectors broadly disseminated among various Gram-negative bacteria. Their capacity for inter-host transmission facilitates the spread of antimicrobial resistance genes across diverse ecological systems, posing a significant threat to global public health. Since their initial identification in 2006 within the multidrug-resistant plasmid pK245 from *Klebsiella pneumoniae*, research on the physiological features, epidemiological characteristics, and specific role of IncR plasmids in resistance dissemination has remained limited and fragmented. To address this gap, the present review systematically examines current literature on IncR plasmids, focusing on their host range, resistance gene composition, coexistence with transposable elements and insertion sequences, and their co-integration with other plasmids (e.g., IncF and IncX3), which often leads to the formation of co-linear plasmids. Available evidence indicates that IncR plasmids are predominantly found in *Enterobacteriaceae* (e.g., *Escherichia coli*, *Klebsiella pneumoniae*, *Enterobacter cloacae*, *Salmonella* spp.) and to a lesser extent in non-fermenters such as *Pseudomonas aeruginosa*. Despite their low copy number, which minimizes the metabolic burden on the host, IncR plasmids demonstrate high stability and retention within bacterial populations, enabling their long-term persistence and global dissemination. In recent years, IncR-carrying strains have been increasingly identified in isolates from human, animal, and environmental sources. Moreover, their co-integration with other resistance plasmids may further expand their resistance spectrum and host range, presenting a growing challenge to clinical antimicrobial therapy. This review aims to summarize the key genetic features and transmission dynamics of IncR plasmids, providing theoretical insights for the control of their global dissemination and guidance for future research into their resistance mechanisms and evolutionary trajectories.

## Introduction

Carbapenems are considered the “last line of defense” against infections caused by multidrug-resistant Gram-negative bacteria. However, their clinical efficacy is increasingly undermined by a global surge in resistance. In recent years, the widespread emergence of carbapenem-resistant *Enterobacteriaceae* (CRE) has posed a significant threat to public health worldwide (Ma et al., 2023). The resistance mechanisms of CRE are complex and multifaceted, involving horizontal gene transfer, protein structural remodeling, and metabolic reprogramming, which together constitute a highly dynamic evolutionary process. This process reflects a combination of adaptive traits (current state adaptations) and the adaptability (evolutionary potential) of bacterial populations, where adaptability facilitates long-term survival and evolution under selective pressure.

Currently, the core resistance mechanisms of CRE can be broadly categorized into four main types: (1) enzymatic hydrolysis, which plays a dominant role—carbapenemases such as KPC-type serine β-lactamases, NDM-type metallo-β-lactamases, and OXA-48-type enzymes hydrolyze the β-lactam ring, rendering carbapenems ineffective. These resistance genes are often located on mobile genetic elements (e.g., IncF plasmids, Tn4401 transposons), enabling cross-species dissemination via conjugative transfer; (2) multidrug efflux pumps (e.g., AcrAB-TolC) that actively expel antibiotics from the cell in an energy-dependent manner; (3) target modification, particularly mutations in penicillin-binding proteins (PBPs) such as those encoded by the mrdA gene, which reduce the binding affinity of antibiotics; and (4) alterations in membrane permeability, including downregulation of porin proteins (e.g., OmpK35/36) and structural modifications of lipopolysaccharides, which collectively hinder antibiotic entry into the cell (Lutgring, 2019).

Notably, these mechanisms often act synergistically, dramatically enhancing resistance phenotypes. For instance, the coexistence of carbapenemase production and efflux pump activation can elevate the minimum inhibitory concentration (MIC) by 64–128-fold, and when coupled with porin loss, resistance can increase exponentially—forming a so-called “enzyme–pump–membrane–target” quadripartite resistance model that significantly compromises the efficacy of conventional combination therapies (Yekani et al., 2022; Mmatli et al., 2020; Allander et al., 2024).

Among these mechanisms, plasmids play a critical role as transferable genetic platforms facilitating interspecies dissemination of resistance determinants. Based on incompatibility (Inc) typing, bacterial plasmids are categorized into several families, including IncF, IncA/C, IncL/M, IncN, IncX3, IncHI2, IncI, IncP, IncT, and IncR. In recent years, IncR plasmids have garnered increasing attention due to their widespread presence in *Enterobacteriaceae* and frequent co-carriage of multiple resistance genes.

A notable feature of IncR plasmids is their non-conjugative nature, as they typically lack a complete conjugation machinery. Nevertheless, they are frequently found in co-residence with self-transmissible plasmids (e.g., IncF and IncX), enabling horizontal gene transfer (HGT) through mobilization. Moreover, IncR plasmids have demonstrated high stability in multidrug-resistant clinical isolates, potentially attributed to their unique replication maintenance systems, stability-associated genes, and abundance of insertion sequences (ISs; Qi et al., 2023).

Given the escalating crisis of antimicrobial resistance (AMR), IncR plasmids have emerged as key vectors for the dissemination of critical resistance genes, such as *bla*_KPC_, *bla*_CTX-M_, and qnr, conferring resistance to carbapenems, cephalosporins, and fluoroquinolones. Their frequent coexistence with multi-replicon plasmids further broadens the resistance spectrum and ecological adaptability of host bacteria, while enhancing their capacity for adaptive evolution in response to environmental pressures—ultimately facilitating long-term persistence (García-Fernández et al., 2009).

Despite existing studies on the molecular epidemiology of IncR plasmids, many aspects of their physiological function, mechanisms of genetic stability, and synergistic dissemination with other plasmids remain poorly understood. Therefore, this review aims to systematically summarize the structural features, resistance gene composition, transmission mechanisms, and potential clinical and public health impacts of IncR plasmids, providing a theoretical foundation for future research and new perspectives for combating antimicrobial resistance.

### Literature search strategy

The publications selected for this review were identified using the keyword “IncR plasmids” as the search term in PubMed. As of March 26, 2025, a total of 230 articles were retrieved.

### Distribution characteristics of IncR plasmids

Since their initial identification in 2006 (Rodrigues et al., 2014), IncR plasmids carrying carbapenemase genes have been widely isolated from human, animal, and environmental samples worldwide, demonstrating historically acquired adaptability and the potential for cross-ecosystem dissemination. Due to their frequent co-existence with other resistance plasmids, IncR plasmids have emerged as critical mediators in the horizontal transfer of antimicrobial resistance genes, posing a growing threat to global public health and signifying a new phase in the dissemination of carbapenem resistance.

**Figure 1:**
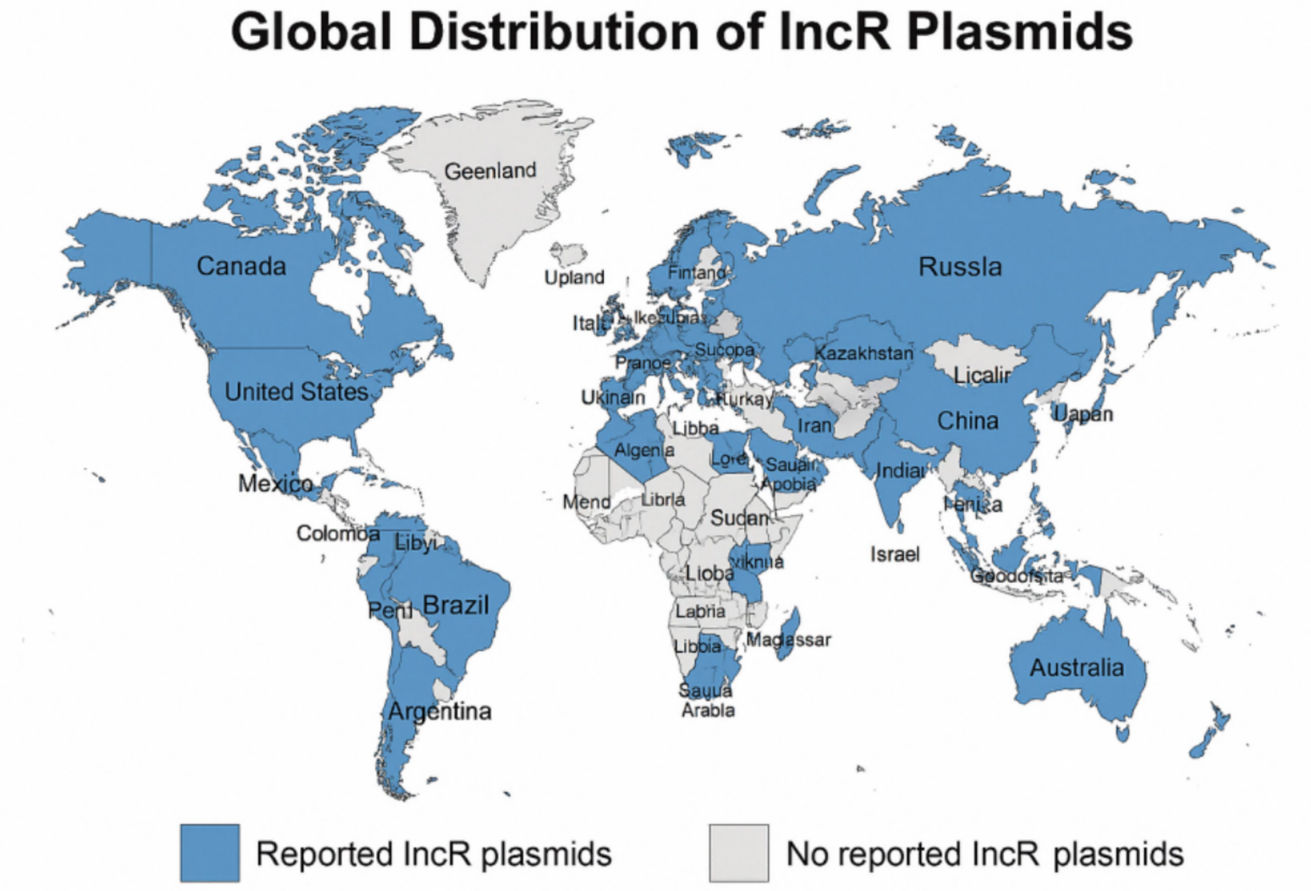
Global distribution map of IncR plasmids.

Studies have shown that bacterial strains harboring IncR plasmids have been detected in a wide range of countries and regions, including China, various European nations (such as Italy, Spain, France, Greece, the United Kingdom, Finland, Portugal, Poland, the Czech Republic, and Switzerland), the Americas (including the United States, Canada, Argentina, Brazil, Mexico, and Ecuador), Asia (including Israel, Japan, Vietnam, India, and Saudi Arabia), as well as North Africa and the Middle East (including Egypt, Libya, and Turkey; Figure 1). Among these, China reports the highest number of studies and documented cases globally, with Henan Province identified as a particularly high-prevalence region (Fang et al., 2024).

Strains carrying IncR plasmids originate from a wide range of sources, broadly classified into clinical and non-clinical categories. Clinical isolates account for approximately two-thirds of reported cases, with sample types including blood, urine, sputum, feces, and wound secretions—covering infections such as bloodstream infections, urinary tract infections, respiratory infections, burn-related infections, and chronic wound infections. The predominant bacterial hosts include *Escherichia coli*, *Klebsiella* spp., and *Enterobacter cloacae*.

Non-clinical samples encompass wild birds, poultry, pork, river water, and wastewater. Studies have highlighted that wild animals may act as important reservoirs for resistant bacteria, thereby facilitating horizontal gene transfer of antimicrobial resistance across ecological boundaries (Qi et al., 2023; Kurittu et al., 2021; Zou et al., 2023; Viana et al., 2020). Consequently, enhanced surveillance of IncR plasmids in non-clinical settings is essential to prevent cross-species transmission and curb the global spread of antimicrobial resistance.

The “One Health” concept underscores the interdependence of human, animal, and environmental health, advocating for multidisciplinary collaboration to address global challenges such as antibiotic resistance (Hidalgo et al., 2013). Existing studies have shown that the dissemination of IncR plasmids across diverse ecological niches aligns closely with this framework. For example, in Spain, an IncR plasmid carrying the 16S rRNA methyltransferase gene armA, along with *bla-*_DHA-1_, *bla*-_SHV-11_, and qnrB4, was first detected in an ST11-type *K. pneumoniae* strain isolated from a pet, suggesting that companion animals may serve as key nodes in the transmission of resistance genes (Hidalgo et al., 2013). Similarly, in *E. coli* isolates from calves, the qnrB19 gene was located on an IncR plasmid coexisting with transposable elements, indicating a high potential for dissemination. Additional research has shown that multidrug-resistant *K. pneumoniae* strains from wild birds often harbor resistance determinants mediated by plasmids highly homologous to those found in clinical isolates, underscoring the potential risk of inter-ecological transmission of resistance (Wang et al., 2023; Pérez-Palacios et al., 2023).

At present, the host range of IncR plasmids continues to expand, encompassing a variety of *Enterobacteriaceae* and *non-Enterobacteriaceae* species. Reported hosts include *K. pneumoniae*, *Citrobacter freundii*, *Escherichia coli*, *E. cloacae, Salmonella* spp., *Morganella* spp., *Providencia* spp., as well as non-fermenters such as *Pseudomonas aeruginosa*, other *Pseudomonas* spp., *Streptococcus mucilaginous*. This broad host adaptability, resulting from both current environmental adaptation and past evolutionary processes, underscores the role of IncR plasmids as key platforms for the cross-species dissemination of resistance genes.

At the molecular epidemiology level, *E. coli* and *K. pneumoniae* strains harboring IncR plasmids exhibit high sequence type (ST) diversity, indicating a strong capacity for dissemination across various host backgrounds. In *E. coli*, ST968 is the most commonly identified, whereas *K. pneumoniae* is predominantly associated with high-risk clones ST11 and ST15 (Qian et al., 2020; Feng et al., 2019). These high-risk clones are not only prominent multidrug-resistant (MDR) lineages of clinical concern but may also serve as “supercarriers” for IncR plasmids, providing a stable genetic platform for interspecies plasmid dissemination. Additionally, ST1023 and other sequence types have been identified in *Salmonella*, further expanding the known host spectrum (Calia et al., 2022).

The observed ST diversity highlights the extensive multi-host adaptability of IncR plasmids within *Enterobacteriaceae*, which includes both the immediate survival advantage in diverse host environments and the potential for long-term evolutionary adaptation. This adaptability suggests their function as recombination and gene integration hubs, playing a critical role in the emergence and evolution of multidrug resistance.

### Structural features of IncR plasmids

IncR plasmids are mosaic MGEs assembled through ongoing module recombination, exhibiting both currently functional adaptive traits and naturally selected broad-host-range transferability. Although most IncR plasmids lack traditional conjugative transfer systems (such as tra operons), studies have shown that they can successfully transfer to a variety of Gram-negative hosts (e.g., *E. coli* EC600 and *K. pneumoniae* ATCC13883) with only moderate metabolic burden (Xiao et al., 2024). This suggests that their transfer may rely on heterologous transposons or co-resident conjugative plasmids to facilitate horizontal gene transfer. Such a cooperative transfer mechanism positions IncR plasmids as crucial players in the inter-host transmission of resistance factors.

Structurally, IncR plasmids consist of a conserved core backbone and highly variable mobile regions. The core region typically includes replication initiation genes (e.g., repB), plasmid stability maintenance factors (e.g., parAB, stb), SOS response-associated genes (e.g., umuCD), IIB class intron reverse transcriptase (retA), polymerase resolving enzymes (resD), and various toxin-antitoxin systems (e.g., vagCD) (Compain et al., 2014). Additionally, some IncR plasmids carry transcriptional regulatory factors that modulate replication rates, thereby enhancing host adaptability in the present context (i.e., current capacity to respond to environmental changes), and maintaining low-copy stability, which may also contribute to long-term evolutionary processes.

In terms of replication regulation, IncR plasmids generally possess a replication origin (oriV) and encode specific replication initiation proteins (e.g., RepA or RepB) to initiate autonomous replication (Jing et al., 2019). Some plasmids regulate copy number and replication frequency via the oriV region, which contains iteron sequences, thereby achieving a dynamic balance between replication efficiency and metabolic burden (Loftie-Eaton and Rawlings, 2010). Although most IncR plasmids lack typical partitioning systems (e.g., par modules), their stability is maintained through interactions with the host chromosome or endogenous toxin-antitoxin systems (e.g., HigBA, ParDE), ensuring stable transmission across cell generations (Qi et al., 2021; Fraikin et al., 2020).

The variable region of IncR plasmids is the main source of their genetic diversity, harboring multiple antibiotic resistance genes (ARGs), insertion sequences (ISs), transposons (Tns), and integrons. Among these, IS26 insertion sequences are particularly common, with individual plasmids carrying six to nine copies (Harmer and Hall, 2016). These sequences are widely involved in mediating the insertion, deletion, and inversion rearrangements of resistance genes, forming complex transposon structures (Harmer and Hall, 2016). The variable regions of different plasmids exhibit high heterogeneity between hosts, reflecting intense recombination-driven and environmentally selected adaptive evolution (i.e., an ongoing process by which plasmids evolve to better fit environmental pressures), which enhances the flexibility and dissemination potential of their resistance gene platforms.

### Antibiotic resistance genes of IncR plasmids and their role in the dissemination of antimicrobial resistance

As key mobile genetic elements, IncR plasmids play a central role in the dissemination of antibiotic resistance among bacterial populations. Studies have shown that IncR plasmids frequently harbor multiple antibiotic resistance genes (ARGs), with particular emphasis on carbapenemase-encoding genes associated with resistance to β-lactam antibiotics. Numerous IncR plasmids reported to date carry a diverse array of resistance genes and often employ co-localization mechanisms to enhance multidrug resistance in bacterial hosts. These plasmids commonly encode various β-lactamase genes in conjunction with genes conferring resistance to other antibiotic classes, including quinolones, aminoglycosides, sulfonamides, and tetracyclines, thereby significantly broadening the antimicrobial resistance spectrum of their host strains. A notable example is the hypervirulent carbapenem-resistant *K. pneumoniae* (CRKP) strain KPN945 isolated in China, in which *bla*_IMP-4_ is located on a transferable IncR plasmid—further underscoring the pivotal role of IncR plasmids in the dissemination of multidrug-resistant bacteria (Xiao et al., 2024).

Beyond the carriage of individual ARGs, IncR plasmids are capable of fusion with other resistance plasmids, accelerating horizontal gene transfer and enhancing the resistance phenotypes of their host bacteria. For instance, hybrid IncN-IncR plasmids have been reported to co-harbor *bla*_TEM-40_, *bla*_KPC-2_, and *bla*_IMP-4_, while IncFII-IncR-IncN fusion plasmids have been found to carry both (Xiao et al., 2024; Sun et al., 2023). The formation of such chimeric plasmids is likely driven by recombination events or genetic rearrangements mediated by insertion sequences such as IS26. These events expand the pathways through which ARGs can disseminate, thereby accelerating the evolution of multidrug-resistant bacterial strains.

Furthermore, a novel FRI-type carbapenemase gene, *bla*_FRI-12_, has been identified in an *Enterobacter asburiae* strain, localized on a ~ 110 kb IncR plasmid and exhibiting high sequence similarity to the previously characterized *bla*_FRI-5_. This finding suggests that the global prevalence of FRI-type carbapenemases may be underestimated and highlights the need for further investigation into their molecular characteristics and resistance mechanisms (Mataseje et al., 2024).

Regionally, the dissemination patterns of IncR plasmids have attracted increasing attention. In China, for example, a *Citrobacter freundii* isolate was found to carry an IncR plasmid, pC275-2, which harbors both *bla*_KPC-2_ and *bla*_NDM-1_. This 46,050 bp plasmid may contribute to the clonal spread of resistant strains in specific geographic regions. Whole-genome sequencing revealed that the *bla*_NDM-1_ region possesses the ability to mobilize between plasmids and chromosomes, while *bla*_KPC-2_ is embedded within a novel genetic context. The distinct gene arrangement of this plasmid may accelerate its dissemination in clinical settings and enhance the bacterial host’s ability to adapt to selective pressures, thereby improving its adaptability to changing environments (Zhou et al., 2025).

It is also noteworthy that IncR plasmids frequently carry resistance genes unrelated to β-lactams, such as the tetracycline resistance gene tetA, sulfonamide resistance genes sul1 and sul2, macrolide resistance genes ermB and mphA, and the 16S rRNA methyltransferase gene rmtB. The coexistence of such diverse ARGs positions IncR plasmids as reservoirs of multidrug resistance, substantially enhancing bacterial survival and competitiveness under antibiotic selection pressure (McMillan et al., 2020).

In *K. pneumoniae* ST11—one of the most prevalent lineages in nosocomial infections—IncFII/IncR fusion plasmids have emerged as major vectors of *bla*_KPC-2_. These plasmids frequently co-harbor *bla*_NDM-5_, *bla*_CTX-M-15_, and *bla*_TEM-1B_, complicating treatment strategies and posing significant clinical challenges. Comparative genomic analyses indicate high sequence conservation of these plasmids within the ST11 clonal group, suggesting stable transmission within clinical bacterial populations and reinforcing their role as crucial mediators of carbapenem resistance in *K. pneumoniae* (Sun et al., 2023).

### Functional characterization of genes encoded on IncR plasmids

Genomic analyses of IncR plasmids reveal that their stable maintenance within host cells relies on multiple highly coordinated functional modules. In terms of replication control, IncR plasmids typically utilize the RepB protein as a central replication initiator, which specifically recognizes and binds to the origin of replication (ori) to recruit the host replication machinery and initiate plasmid DNA synthesis. RepB generally harbors characteristic DNA-binding domains and ATP-binding motifs, indicating its critical role in initiating replication and regulating replication frequency. By maintaining an optimal plasmid copy number, RepB ensures long-term plasmid stability without imposing a significant metabolic burden on the host (Jing et al., 2019).

For orderly plasmid segregation, IncR plasmids commonly carry the canonical partitioning system composed of parA and parB. The ParA protein, a member of the P-loop ATPase family, facilitates plasmid positioning and dynamic distribution within the cell through ATP hydrolysis, while ParB binds to specific parS sites to form stable DNA-protein complexes that anchor plasmids for ParA-mediated localization. This partitioning system ensures accurate inheritance of plasmids by daughter cells during host cell division, thereby enhancing intergenerational stability. This mechanism is especially critical for non-conjugative plasmids like IncR, which lack self-transfer capacity (Compain et al., 2014; Volante and Alonso, 2015).

In addition, IncR plasmids exhibit considerable recombination potential, which contributes to their structural plasticity and adaptive evolution, where ‘adaptive’ refers to the evolutionary process through which these plasmids are capable of adjusting to environmental pressures over time, ultimately reflecting the outcome of natural selection. For instance, the rfsF-resD site-specific recombination system can mediate gene module insertion, deletion, or rearrangement, facilitating the rapid acquisition of novel functional elements under fluctuating environmental conditions. The retA gene, which encodes a group IIB reverse transcriptase, may act in concert with mobile genetic elements to mediate the mobility of self-splicing introns, further enriching the genetic diversity of IncR plasmids (Jing et al., 2019).

Although genes such as repB, parAB, and vagCD do not directly encode antibiotic resistance determinants, they play essential roles in ensuring plasmid stability and transferability. By doing so, they indirectly facilitate the long-term persistence and widespread dissemination of resistance genes. The synergistic interactions among these functional modules form the molecular basis for the evolutionary adaptability, where ‘evolutionary adaptability’ refers to the ability of IncR plasmids to adjust to environmental pressures over time, ultimately reflecting the outcome of natural selection, and resistance propagation capacity in bacterial populations (Calia et al., 2022).

### Cointegrate plasmids associated with IncR elements

Cointegrate plasmids are composite replicons formed via the fusion of two or more independent circular plasmids within the same host cell, mediated by homologous recombination or transposition events. This process typically depends on mobile genetic elements such as insertion sequences (e.g., IS26), transposons (e.g., the Tn3 family), and host-encoded RecA-dependent homologous recombination pathways. Among these, IS26 is one of the most prevalent and active recombination mediators, playing a central role in the fusion of IncR plasmids with other replicons. When both plasmids harbor IS26 elements, their inverted repeat (IR) sequences can initiate strand exchange reactions, forming a Holliday junction-like intermediate, and with the participation of RecA, complete recombination to generate a stable cointegrate structure. This mechanism facilitates genetic integration and provides a molecular basis for IncR plasmids to enter the network of mobile genetic elements and participate in horizontal dissemination of resistance genes (Smillie et al., 2010).

Beyond their structural recombination potential, IncR plasmids also serve as platforms for resistance gene amplification. In the ST11 clone of carbapenem-resistant hypervirulent *Klebsiella pneumoniae* (CR-hvKP), a cointegrate plasmid designated pKPC-CR-HvKP4-SH9 has been reported to carry up to five copies of the *bla*_KPC-2_ gene. These copies are located within a 5.7 kb tandem repeat unit flanked by IS26 elements, forming a composite transposon structure resembling the non-Tn4401 element NTE-KPC-Id, and may further generate circular translocatable units (TUs). Such structures can be inserted between other IS26 elements through transposase Tnp26 activity or homologous recombination, enabling high-frequency amplification of *bla*_KPC_ genes. This finding highlights the structural advantages and dynamic adaptability, where ‘dynamic adaptability’ refers to the ability of IncR plasmids to rapidly adjust to changing environmental pressures, thereby facilitating the preservation and amplification of resistance determinants (Aihara et al., 2024; Dong et al., 2019).

Although most IncR plasmids lack complete conjugative machinery, their fusion with mobilizable replicons to form chimeric structures may confer passive mobilization potential. For instance, a strain of *Klebsiella acidophilus* (designated *K. ascorbata* L4110) was recently identified, and its complete genome sequence has been deposited in GenBank (BioSample accession: SAMN44237076). Carrying a *bla*_NDM-1_-bearing plasmid with a typical IncR-IncFIA (HI1) chimeric backbone, isolated from a fecal sample of an outpatient (Table 1). This plasmid lacks full-length relaxase and T4SS genes and is thus classified as a non-conjugative plasmid. To assess its transferability, the plasmid was transformed into *E. coli* EC600, generating the transformant L4110hy-EC600. Conjugation assays showed that the plasmid could not self-transfer, suggesting its mobility may rely on co-resident conjugative plasmids or heterologous T4SS machinery, classifying it as a “dependently mobilizable plasmid.” Furthermore, the transformant exhibited significantly elevated minimum inhibitory concentrations (MICs) against various carbapenems and cephalosporins (e.g., meropenem MIC ≥ 2 μg/mL, ceftazidime MIC > 128 μg/mL), whereas the parental EC600 strain remained susceptible, confirming that stable expression of *bla*_NDM-1_ is the primary driver of the resistant phenotype (Table 2).

**Table 1:** Genomic characterization of L4110hy.

Plasmid	Total number of bases (bp)	G + C content (%)	Plasmid replicon type	Resistance genes	Virulence factors
*K. ascorbata* strain L4110hy 1	4,845,762	54	NA	*bla*_CTX-M-77_	nlpI
*K. ascorbata* strain L4110hy 2	301,733	48	Col440I, IncFIB(K), IncHI1A, IncHI1B(R27)	/	clpK1、terC
*K. ascorbata* strain L4110hy 3	52,121	52	IncFIA(HI1), IncR	*bla*_NDM-1_	/
*K. ascorbata* strain L4110hy 4	4,019	46	NA	/	/
*K. ascorbata* strain L4110hy 5	2,723	50	Col440I	/	/
*K. ascorbata* strain L4110hy 6	2,206	46	NA	/	/
*K. ascorbata* strain L4110hy 7	1,554	47	NA	/	/

**Table 2:** Results of drug sensitization experiments.

Antibiotics	MIC value (mg/L)		
	L4110hy	L4110hy-EC600	EC600
Aztreonam	0.5	0.5	0.06
Imipenem	2	2	0.125
Meropenem	2	2	0.06
Ceftriaxone sodium	64	128	0.125
Cefotaxime sodium	128	128	0.125
Ceftazidime	64	>128	0.5
Levofloxacin	0.25	0.25	0.06
Ciprofloxacin	0.25	0.25	0.008
Amikacin	4	2	1
Gentamicin	1	1	1
Piperacillin sodium and tazobactam sodium	128	128	4
Fosfomycin + glucose 6-phosphate	1	1	1
Chloramphenicol	32	32	8
Trimethoprim-sulfamethaxazole	≤0.125	≤0.125	≤0.125
Amoxicillin/clavulanic acid	64	8	8
Cefepime	8	8	0.06
Ceftazidime/avibactam	>128	>128	0.25
Tigecycline	0.5	0.5	0.125

Of particular interest, certain IncR plasmids have acquired a complete oriT–mobC mobilization module through structural recombination, granting them the capacity to recognize the origin of transfer and participate in DNA nicking and transfer initiation. Although these plasmids still lack a complete conjugation system, they can be mobilized via the conjugative machinery of co-resident plasmids, significantly enhancing their interspecies transmission potential. For example, the pKPN945B plasmid contains genetic modules derived from multiple replicon types, including a complete oriT–mobC mobilization system. The oriT region originates from the R46 plasmid family, and the MobC protein can bind to the nick site, assisting in the initiation of plasmid DNA processing and transfer. Additionally, this plasmid encodes the RepE replication initiator protein, typically associated with IncF replicons, in place of the canonical IncR-encoded RepB, indicating a diversification of replication mechanisms via replicon fusion. These “atypical” IncR plasmids challenge the traditional view of their non-mobilizability and underscore their remarkable structural plasticity and potential for dissemination, providing novel molecular pathways for the spread of multidrug resistance genes (Xiao et al., 2024; Coupland et al., 1987).

## Conclusion

IncR plasmids exhibit high genetic stability and robust maintenance in bacterial hosts, with a structurally flexible and highly adaptive architecture, where ‘adaptive’ refers to the ability of IncR plasmids to adjust to environmental pressures over time, facilitating their persistence and evolution in diverse conditions. They are capable of harboring a broad spectrum of resistance genes, particularly those conferring resistance to commonly used antibiotics such as β-lactams, quinolones, and aminoglycosides. Their widespread occurrence across clinical, environmental, and animal-associated microbial populations underscores the substantial public health threat posed by the potential for global dissemination of the resistance determinants they carry (Compain et al., 2014; Sheppard et al., 2016).

Looking ahead, future research on IncR plasmids should focus on the diversity of their genetic structures, the multiplicity of their resistance mechanisms, and the complexity of their dissemination modes. Special attention should be paid to their ecological behavior and distribution in both clinical and natural environments. Moreover, in-depth investigation into cointegration events between IncR and other plasmids is essential to unravel the underlying molecular mechanisms facilitating the spread of resistance traits, with the ultimate goal of identifying key determinants of transmissibility (Partridge et al., 2018).

From a public health perspective, there is an urgent need for precise surveillance of IncR plasmids, the development of novel strategies to inhibit resistance gene expression, and the exploration of cutting-edge technologies such as gene editing for resistance control. Only through interdisciplinary collaboration—integrating genomics, molecular biology, clinical medicine, and public health—can we effectively curb the global spread of IncR plasmids and the resistance genes they bear. Continued and focused research into their functions and mechanisms is essential to mitigating the threat antibiotic resistance poses to global health.
